# Microbiome dynamics in the congregate environment of U.S. Army Infantry training

**DOI:** 10.1128/spectrum.00474-25

**Published:** 2025-12-17

**Authors:** Car Reen Kok, Michael D. Morrison, James B. Thissen, Shalini Mabery, M. Leigh Carson, Jeffrey A. Kimbrel, Jason W. Bennett, David R. Tribble, Eugene V. Millar, Katrin Mende, Nicholas A. Be

**Affiliations:** 1 Lawrence Livermore National Laboratory 4578, Livermore, California, USA; 2 Department of Preventive Medicine and Biostatistics, Infectious Disease Clinical Research Program, Uniformed Services University of the Health Sciences 1685, Bethesda, Maryland, USA; 3 Henry M. Jackson Foundation for the Advancement of Military Medicine Inc, Bethesda, Maryland, USA; 4 Walter Reed Army Institute of Research 8394, Silver Spring, Maryland, USA; 5 Brooke Army Medical Center, Joint Base San Antonio, San Antonio, Texas, USA; National Center for Genetic Engineering and Biotechnology, Khlong Luang, Pathum Thani, Thailand

**Keywords:** microbiome, metagenomics, military training, infection diagnostic, microbial genomics, military medicine, nosocomial pathogen

## Abstract

Within military training and operational environments, individuals from diverse backgrounds share common spaces, follow structured routines and diets, and engage in physically demanding tasks. While there has been interest in leveraging microbiome features to predict and improve military health and performance, the longitudinal convergence of microbiomes in such constrained environments has not been established. To assess the degree of microbiome convergence, we performed shotgun metagenomic sequencing on swab samples from a military trainee cohort. Samples were taken across four different body sites, three timepoints, and two spatially distinct platoons. We observed evidence of convergence in one platoon, whereby similarity in microbiome composition increased over time, with numerous differentially abundant species. We found no indication of strain transfer between individuals, suggesting that convergence was influenced by external environmental factors, diet, and lifestyle. Microbial shifts observed in the convergence process included a decrease in fungal species, such as *Malassezia restricta* in nasal cavities, and a decrease in *Prevotella* species at inguinal regions across time. Shifts in multiple *Corynebacterium* species were also observed with varying magnitudes depending on the body site. Overall, we provide preliminary evidence of convergence of host microbial communities in military-associated environments that were distinguishable using shotgun metagenomic sequencing approaches. The data presented here on microbiome convergence, dynamics, and stability may inform risk-based mitigation in congregate military settings facilitating development of targeted microbial, dietary, or other interventions to optimize health and performance of military populations.

**IMPORTANCE:**

Microbiome convergence in deployed environments could impact the health and readiness of the warfighter, with potential implications for susceptibility to biothreats. This study describes a shotgun metagenomic approach used to study the microbiomes of swab samples collected at different body sites in a military trainee cohort. The results presented here provide a foundation for developing future microbiome-based interventions and protocols to enhance operational readiness.

## INTRODUCTION

Military training imposes uniquely strenuous demands on the physical and psychological health of soldiers. Trainees operate in an environment at elevated risk for musculoskeletal injury (1), heat exhaustion (2), and infection (3), all of which can interrupt and substantially hinder training cycles. Complications, such as skin and soft-tissue infections, are observed at elevated levels in military trainees, compromising operational readiness for active-duty service members (4). The capacity to predict the likelihood and prevent the incidence of such complications would broadly improve health and readiness of military service members.

There are numerous physical and cognitive metrics for assessing military health and readiness, including body composition, physical performance, cardiovascular metrics, and cognitive and social functioning (5–7). One component of human physiology that has not been explored in depth, with respect to risk stratification for military medicine, is the content and stability of the host microbiome. An abundance of studies now demonstrates that features of the microbiome are clear indicators of clinical outcomes, including in gastrointestinal (GI) health (8, 9), cancer (10, 11), neurological disease (12, 13), and immune dysfunction (14, 15). Our previous efforts show that microbe-relevant signatures differentiate outcomes in military injuries (16–18). We also demonstrated the presence of putative wound infecting species on the uniforms and gear of military trainees (19). Human microbiomes may also exert an impact on the performance and fitness of their hosts, as distinctions have been observed in the gut microbiomes of elite athletes (20, 21).

The composition of the human microbiome demonstrates substantial inter-individual variability; however, microbiomes have been shown to converge between individuals and environments in congregate settings. U.S. Air Force cadets from distinct geographical locales develop convergent microbiomes with environmental surfaces despite their initially diverse microbial content (22). Community coalescence is observed in animal settings, where convergence occurs between individuals and between an individual and the environment (23). Furthermore, several studies have highlighted the influence of the environment and other lifestyle factors on the human gut, ocular, and skin microbiomes (24–26). A previous study on the International Space Station demonstrated the interconnectivity of the human microbiome and the environment under constrained operational conditions (27).

Given the expansive body of literature indicating that microbiome features are predictive of, and/or influential toward, health and performance, the potential transmission or convergence of these features is of substantial interest. Military trainees arrive from distinct national locations, after which they experience both spatially constrained living conditions and highly consistent physical activities and diets. Convergence or transmission of microbiomes between individuals during training could be critically informative for anticipating health and performance risks, ultimately informing potential interventions that could limit adverse outcomes.

U.S. Army Infantry training at Fort Benning, Georgia, is a 14 week period with intensive physical and mental demands, held in a uniquely controlled environment. A previous study (3) enrolled a closed cohort of U.S. Army Infantry trainees over the duration of a training cycle, with longitudinal sampling via skin swabs across multiple body sites. Previous assessments in such trainees employed 16S rRNA sequencing to assess genera distinctions in microbial constituents between subgroups, demonstrating the value of examining microbiome dynamics in a military training environment (3, 28, 29). The current study employed whole metagenome sequencing to further evaluate species-level and non-bacterial constituents in samplings across multiple body sites (nasal, oral, inguinal, and perianal) and collected timepoints from two distinct platoons, housed in spatially separate barracks.

To assess the potential transmission and/or convergence of microbiome features that may influence trainee health, this study examined the microbiome content of surface swabs across multiple timepoints, groups of individuals, and body sites. This assessment sought to address whether microbiome transmission or convergence between individuals is distinguishable over time in a training cohort and whether these observations are specific to a given platoon (i.e., congregate sub-group), or general to all individuals in the training regimen. These analyses assessed the utility of tracking microbial factors for predicting the health, readiness, and performance of future military service members.

## RESULTS AND DISCUSSION

### Microbial abundances of distinct host body sites

Swab samples were collected across four body sites (nasal; NR, oral; OR, inguinal; IR, and perianal; PR) at three timepoints; day 0 (enrollment), day 28, and day 90 (immediately following in-field training) from two platoons with eight trainees per platoon included in this study. Microbiome profiles were generated from shotgun metagenomic sequencing of 171 samples to assess microbial composition along with spatial and temporal shifts and/or convergence during military training.

Genomic content was successfully obtained from low biomass sample swabs across body sites, adequate for shotgun metagenomic sequencing. Sufficient microbial reads (≥1,000) were generated for 91.81% of samples (157/171 samples) for downstream analyses (Table 1). This highlights the utility of swab samples as a quick and non-invasive sample collection method, allowing for future screening of microbiome profiles from similar cohorts.

**TABLE 1:** Number of samples stratified according to platoon, body site, and timepoint

Platoon	Body site	No. of samples		
		Day 0	Day 28	Day 90
Platoon 2	IR	7	6	5
	NR	7	7	7
	OR	4	4	3
	PR	7	7	6
Platoon 4	IR	7	6	7
	NR	5	5	8
	OR	8	8	7
	PR	7	8	8

**Fig 1:**
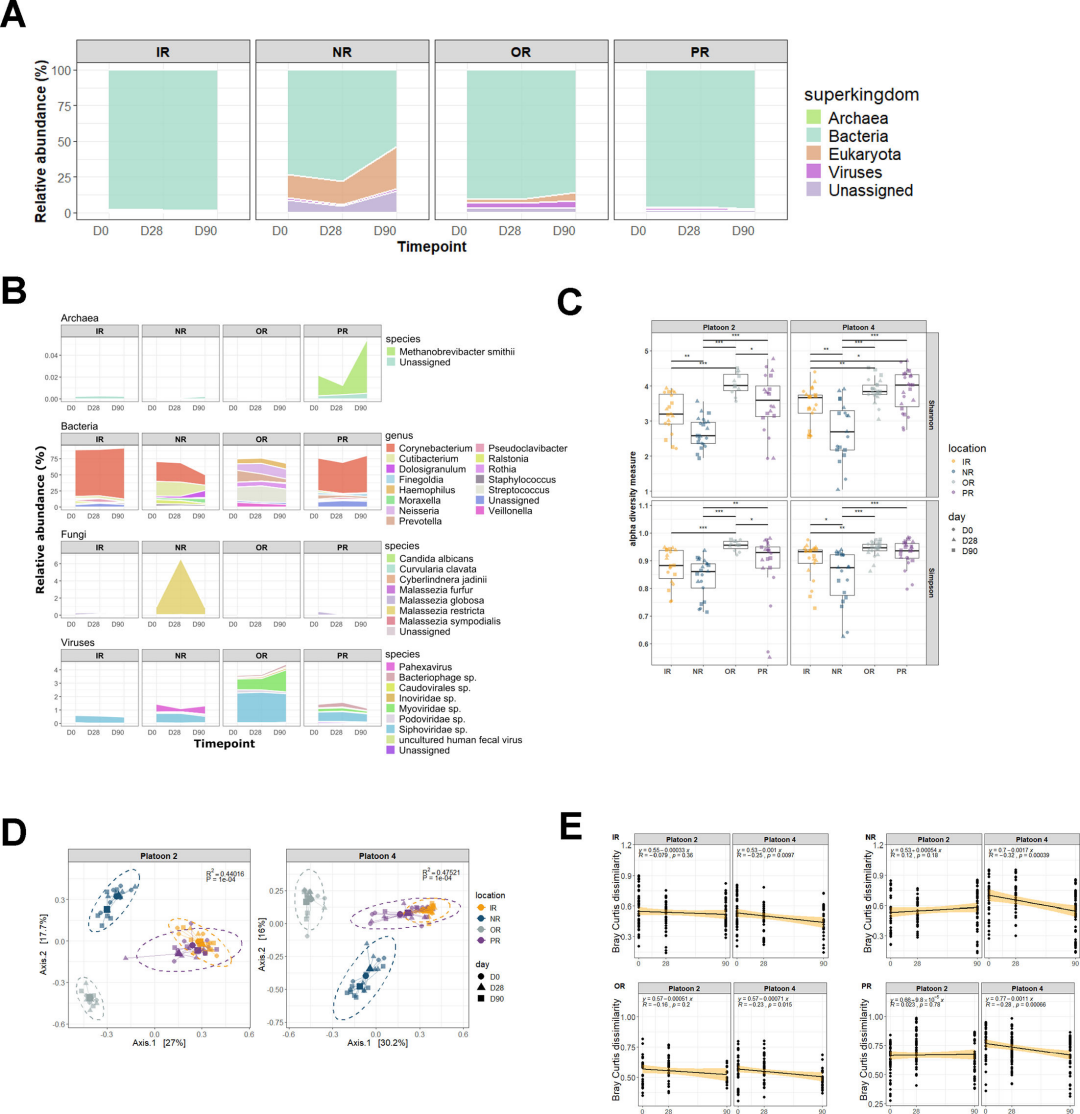
Microbiome composition and diversity across inguinal (IR), nasal (NR), oral (OR), and perianal (PR) samples. Mean relative abundances are represented by stacked bar plots at the (**A**) superkingdom and (**B**) genus/species level. Genus/species representations are arranged from top to bottom according to archaea (≥0.01% relative abundance), (bacteria (≥1% relative abundance), fungi (≥0.01% relative abundance), and virus (≥0.01% relative abundance). (**C**) Microbial alpha diversity was determined using Shannon and Simpson diversity indices. (* adjusted *P*-value < 0.05, ** adjusted *P*-value < 0.01, *** adjusted *P*-value < 0.001). (**D**) Principal coordinates analysis (PCoA) of Bray Curtis dissimilarity distances was used to examine differences in microbiome composition. Each point represents a sample and is colored according to body site and shaped according to timepoint. (**E**) Linear regression of pairwise Bray Curtis dissimilarity distances was used to investigate microbiome convergence over time within platoons.

At the superkingdom level, the samples were composed of predominantly bacterial species (IR; 98.37% ± 1.7%, NR; 67.42% ± 27.84%, OR; 89.07% ± 8.28%, PR; 96.54% ± 3.81%), followed by eukaryotes (IR; 0.52% ± 1%, NR; 21.32% ± 18.8%, OR; 3.66% ± 5.17%, PR; 0.36% ± 0.81%), viruses (IR; 0.67% ± 0.57%, NR; 1.3% ± 1.57%, OR; 3.97% ± 2.36%, PR; 1.42% ± 1.91%) and archaea (IR; 0.005% ± 0.003%, NR; 0.001% ± 0.003%, OR; 0.0005% ± 0.0006%, PR; 0.03% ± 0.097%) (Fig. 1A). The fraction of classified eukaryotic reads in the NR samples consisted mainly of host reads that remained after *in silico* depletion along with fungal *Malassezia* species (Fig. S1A). The overall low abundance of archaeal species aligns with previous reports indicating its rare presence on the mammalian skin microbiome (Fig. 1A) (30). At the genus level, microbial profiles were distinct across sampling sites (Fig. 1B).

To facilitate interpretation of inter-kingdom microbial relative abundances, bacteria genera with ≥1% relative abundance were assessed, while a threshold of ≥0.01% relative abundance was applied to fungal and viral species (Fig. 1B). *Corynebacterium*, *Cutibacterium,* and *Staphylococcus* were found in IR, NR, and PR samples. The observation of multiple *Staphylococcus* species across body sites is consistent with previous observations in such samples, and supports the potential utility of microbiome sampling for risk factors associated with skin and soft-tissue infections in trainees, who are disproportionately impacted by such events (4). *Corynebacterium* species, in particular, are known to be ubiquitous to the human microbiome and are found in high abundance on the skin and nasal cavity (31).

The detection of site-specific genera was also apparent. The PR samples harbored gut-associated genera (e.g., *Bacteroides* and *Faecalibacterium* (32–34)), while the nasal-specific genus *Dolosigranulum* was found in only NR samples (35). In contrast, OR samples were more diverse and showed appreciable detection of different genera, including *Haemophilus*, *Neisseria*, *Rothia,* and *Fusobacterium*.

The NR samples had the highest abundance of fungal species, which mainly consisted of *Malasezzia restricta* and *Malassezia globosa. Candida albicans* and *M. globosa* were also detected in IR and PR samples. Fungal constituents are of particular concern following battlefield injuries, as they can result in invasive wound infections that substantially complicate downstream treatment and rehabilitation (36, 37). Although the fungal species detected here have not been implicated in such injuries, *C. albicans* is observed as a colonizer in burn injuries (38), chronic wounds (39), and wound infection (40), and *M. restricta* is associated with specific bacterial populations in chronic wounds (41). Observation of multiple fungal species here further demonstrates an advantage of the shotgun metagenomic approach for assessing non-bacterial infectious risk factors.

The viral content detected in the samples largely consisted of *Caudovirales* bacteriophages (*Siphoviridae*, *Podoviridae*, *Myoviridae*) with *Siphoviridae* present in all samples and *Myoviridae* largely present in OR samples, aligning with previous findings of the human phageome (42–45). *Pahexavirus,* which contains lytic *Cutibacterium acnes* phages (46, 47), was also present in NR samples. *Methanobrevibacter smithii* was the most abundant archaeal species detected, observed mostly in PR swabs which served as the closest proxy to fecal swabs. *M. smithii* is a methanogen commonly found in the human gut and has been implicated as a potential health marker in disorders such as non-alcoholic steatohepatitis and irritable bowel syndrome (48, 49). Overall, the detected microbial species accurately reflected the expected communities for corresponding sites, further demonstrating the feasibility of using similar swab sampling protocols for accurate representation and broad detection of potential infectious risk factors in the body sites under study.

### Microbiome diversity and composition

Microbial diversity was further evaluated using alpha and beta diversity measures, and comparisons were made across host body sites, platoons, and timepoints. Alpha diversity was calculated using Shannon and Simpson indexes to capture species evenness and richness (Fig. 1C). The OR samples demonstrated significantly higher diversity (*P* < 0.05) compared to both IR and NR samples, while PR samples were significantly more diverse compared to NR samples across both platoons. This observation reflects the rich diversity of the oral microbiome, considered the second most diverse human microbiome community after the gut, with various species colonizing hard teeth surfaces and soft mucosal tissues (50, 51). These results further demonstrate significant differences in microbiome composition between body sites. No significant differences in alpha diversity were observed between timepoints and between platoons regardless of body site (Fig. S1B and C).

Principal coordinates analysis (PCoA) of Bray Curtis distances was used to evaluate similarity in compositional structure between samples, and PERMANOVA analyses were carried out to determine significant compositional differences between factors of interest. PERMANOVA analyses indicated significant effects of body sites, timepoints, and platoons on microbiome composition (Table 2) with body site having the largest sum of squares, therefore accounting for most of the compositional variance. Significant interaction between body site and timepoint was also observed, indicating that the microbiome composition at each body site was influenced by training duration.

**TABLE 2:** Results of 3-factor PERMANOVA analyses based on Bray Curtis distances^*a*^

Factors	Degrees of freedom	Sum of squares	*R* ^2^	*F*	Pr > F
Platoon	1	0.737	0.01357	3.9104	0.0013
Body site	3	23.813	0.43809	42.0891	9.99e−5
Day	2	0.935	0.01720	2.4782	0.0034
Platoon*body site^*b*^	3	0.843	0.01550	1.4891	0.0624
Platoon*day	2	0.365	0.00672	0.9680	0.4674
Body site*day	6	1.809	0.03328	1.5985	0.0085
Platoon*body site*day	6	0.772	0.01420	0.6820	0.9688

^*a*^ The analysis was carried out with 10,000 permutations. Significant factors (platoon, location, and day) were determined alongside any significant interactions.

^*b*^ * represents interactions between the factors.

Given the significant influence of body site on microbiome composition, additional analyses were carried out to determine the effect of timepoint and platoon within each body site (Fig. S1D). Here, a significant effect of timepoint was only observed in NR samples (*P* = 0.003), and no significant interactions between timepoint and platoon were observed in any comparisons. These findings are constrained by the number of samples per timepoint per body site, thus it is possible that additional sampling could strengthen significance in some comparisons.

### Microbiome convergence across time

Given the statistically significant compositional differences between platoons described above (Table 2), microbiome convergence was evaluated using a linear regression of pairwise Bray Curtis dissimilarity distances over time per platoon (Fig. 1E). Significant negative Pearson correlation coefficients (*P* < 0.05) were observed in platoon 4 samples regardless of body site, indicating increased compositional similarity from days 0 to 90. Platoon 2 samples, however, did not display similar trends, with no observable significant changes in Bray Curtis dissimilarity distances over time in any of the sampled body sites. This observation of variable degrees of convergence between subgroups is consistent with previous 16S rRNA-based assessments of oral and nasal microbiota in trainee cohorts and may be dependent on other metadata variables such as interindividual variability and geographic origin (3, 28). This combined body of results indicates that microbiome convergence is not universally observed in congregate settings and is likely dependent on a range of factors, including individual origin and duration of congregate confinement.

**Fig 2:**
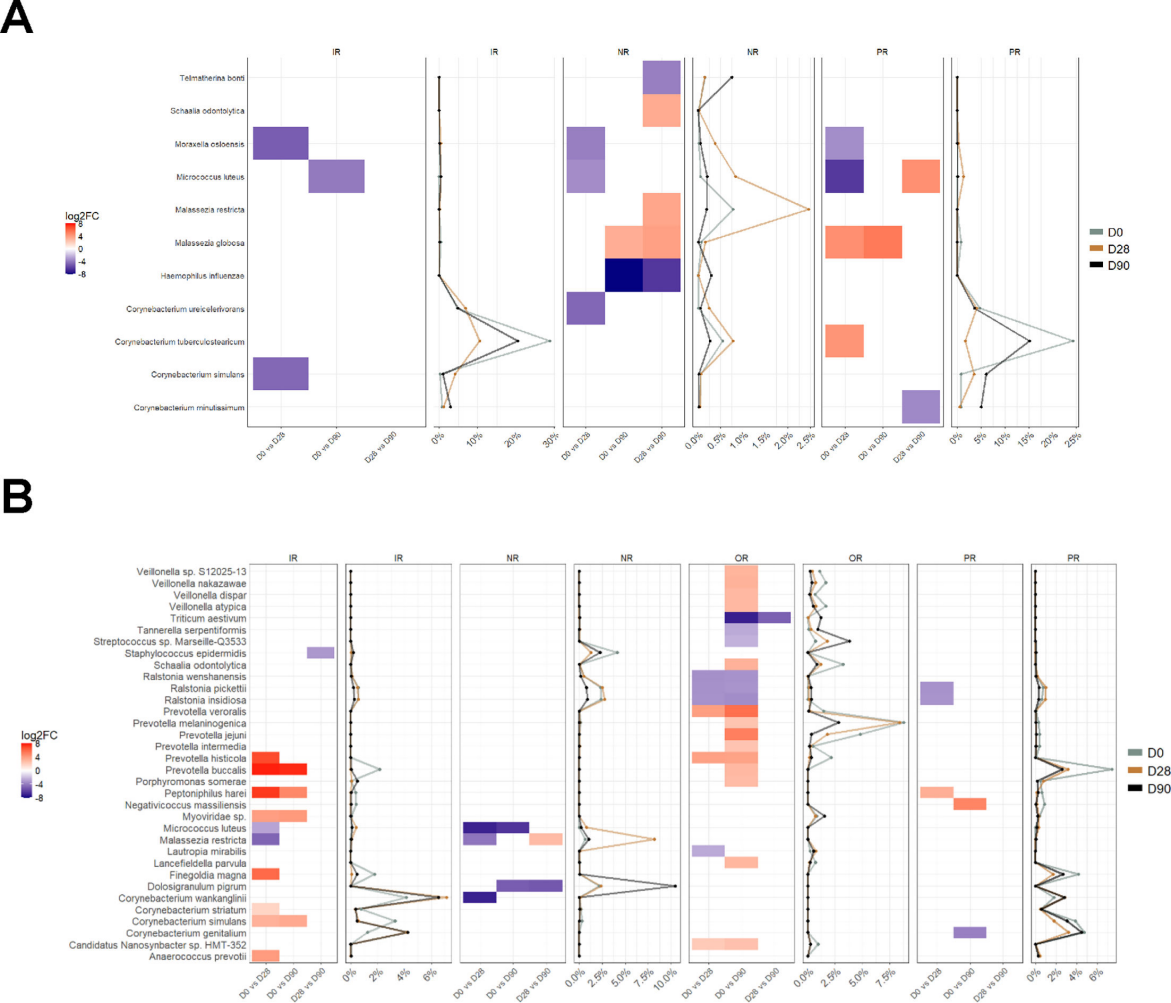
Differentially abundant species between timepoints across inguinal (IR), nasal (NR), oral (OR), and perinanal (PR) samples in (**A**) platoon 2 and (**B**) platoon 4 samples. For every comparison (ie D0 vs D28), red cells represent species that are higher in abundance in the first group (i.e., D0) and blue cells represent species that are higher in abundance in the second group (i.e., D28). The average relative abundances of corresponding species within a body site are visualized as line plots adjacent to the differential abundance plots.

Differential abundance analyses were carried out to identify species potentially involved in the convergence process. Overall, a lower number of differentially abundant species were observed in platoon 2 (Fig. 2A) compared to platoon 4 (Fig. 2B). An increase in *Micrococcus luteus* at later timepoints compared to day 0 was observed in IR and NR samples across both platoons. Changes in *Corynebacterium* species were observed in both platoons with species-specific shifts differing depending on the body site.

In platoon 2, a consistent increase in *Moraxella osloensis* was observed on day 28 compared to day 0 in IR, NR, and PR samples while an increase in *Malassezia globosa* from day 0 to days 28 and 90 was observed in NR and PR samples. A higher abundance of *Haemophilus influenza* was also observed at Day 90 in NR samples. There were no differentially abundant species detected in platoon 2 OR samples. In platoon 4, significant temporal shifts in multiple species were observed across all four body sites. This includes the decrease of several *Prevotella* and *Veilonella* species and an increase in *Ralstonia* species in OR samples with most changes occurring between days 0 and 90.

Alternatively, most of the significant shifts in platoon 4 IR samples were between days 0 and 28, including decreases in *Prevotella* and *Corynebacterium* species. Interestingly, an increase in *Malasezzia restricta* was observed in platoon 4 NR samples from day 0 to day 28, followed by a subsequent decrease from day 28 to day 90. Therefore, while changes in microbiome composition and diversity through time were minimal and involved only a subset of species, our results show evidence of microbiome convergence that was platoon dependent. This could be influenced by platoon-specific routines affiliated with cohabitation or interindividual distinctions between platoons.

While factors such as body region, biological sex and age, and hygiene practices have been shown to strongly influence skin microbiome composition, the effects of cohabitation on the skin microbiome have also been established (52, 53). In a study with 159 individuals, Song et al. (53) observed increased similarity in the skin microbiomes of cohabitating individuals and between individuals and their pets compared to non-cohabitating individuals. Furthermore, in a 5-month long microbiome analysis of a cohort of United States Air Force Academy cadets, Sharma et al. (22) observed evidence of greater skin microbiome similarity in cohabitating roommates. Similarly, Zhou et al. (54) reported significantly less variation in stool, oral, skin, and nasal microbiomes between individuals from the same household compared to between unrelated individuals. In addition, the authors described higher stability of stool and oral microbiomes compared to skin and nasal microbiomes in healthy individuals, likely a result of direct interaction between skin and nasal regions with the environment.

Household-specific microbial communities and microbiome exchange between the home environment and humans have also been previously discussed (55, 56). The microbial exchange and homogenization that occur within a household is likely sourced from direct and frequent contact between individuals and from the surrounding built environment. This homogenization is likely enhanced in military barracks, whereby trainees are forced to live in close proximity with each other, including the use of shared common areas and sleeping quarters, along with adopting similar diet and lifestyles during the training period.

### Strain tracking between and within individuals

**Fig 3:**
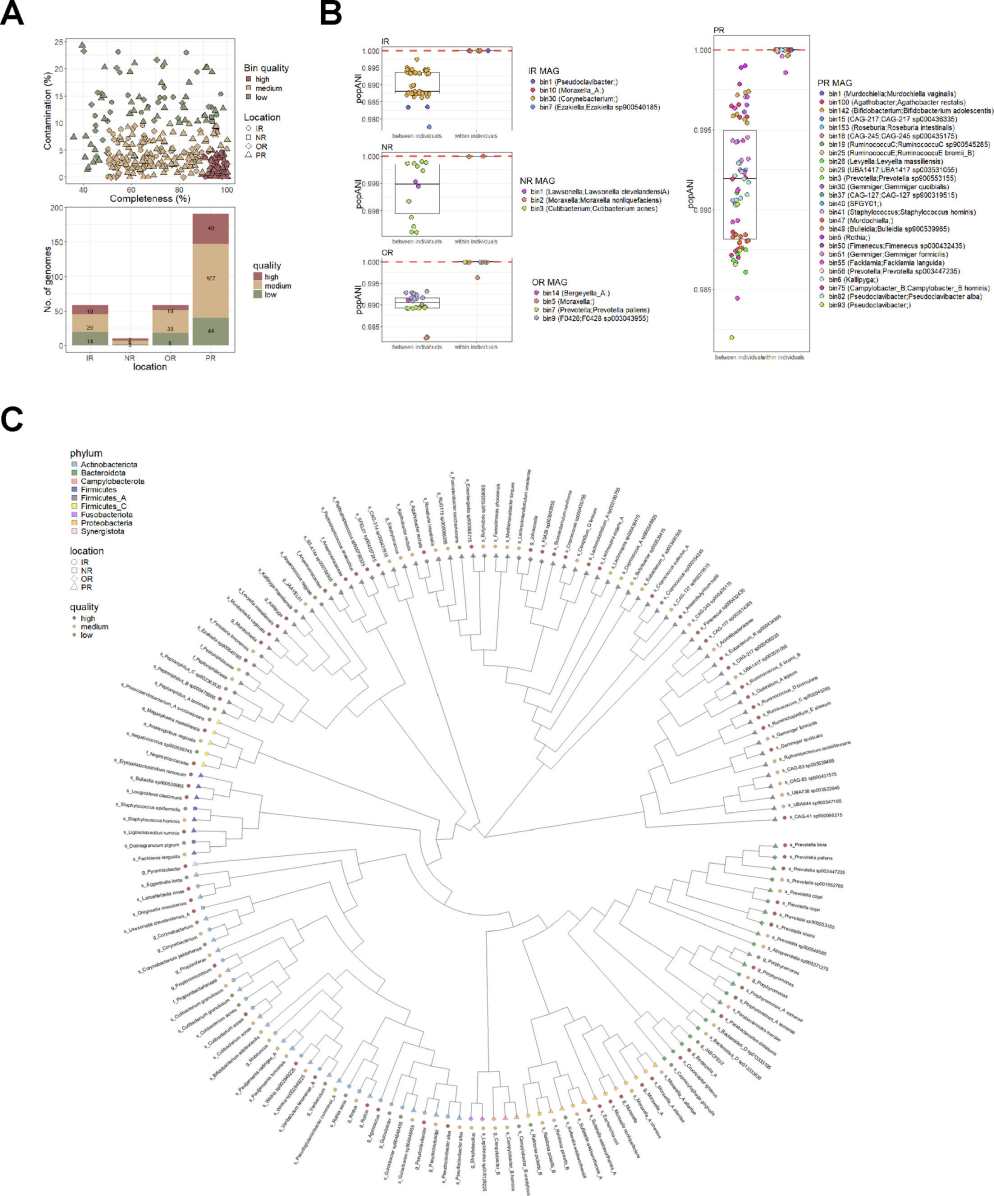
Metagenome assembled genomes (MAGs) and strain-level comparisons. (**A**) Metagenome co-assemblies were carried out within each body site, resulting in 69 high-quality (completion ≥ 90% and contamination ≤ 5%), 171 medium-quality (completion ≥ 50% and contamination ≤ 10%), and 69 low-quality MAGs (completion < 50% or contamination > 10%). (**B**) Pairwise popANI values were used to determine strain transfer between individuals. A threshold of 99.999% (red dotted lines) was used to determine identical strains. Between-sample comparisons were only carried out when genomes had ≥50% coverage breadth. (**C**) Cladogram representing all MAGs that were recovered from each co-assembly. Each leaf in the cladogram represents a MAG whereby taxonomy was assigned according to GTDB-Tk. Each leaf is colored according to phyla and shaped according to the body site whereby the MAG was derived from. Outer colored circles represent bin quality.

To facilitate the analysis of strain-level exchange between trainees, a single-nucleotide polymorphism-based comparison of metagenome assembled genomes (MAG) was carried out using inStrain (57). Assemblies were carried out per body site, and 69 high-quality (completion ≥90% and contamination ≤5%) and 171 medium-quality (completion ≥50% and contamination ≤10%) MAGs were generated (Fig. 3A), resulting in a phylogenetically diverse set of genomes (Fig. 3C). The assembly of PR samples resulted in the highest number of MAGs and, subsequently, the highest number of high-quality MAGs. This was likely a result of PR samples having the highest number of sequenced reads. For every sample, reads were mapped against all 240 medium and high-quality MAGs, and pairwise popANI (population Average Nucleotide Identity) values were generated per genome. Strain-level comparisons were only carried out between samples for genomes with at least 50% coverage, with a threshold of 99.999% popANI indicating identical strains (Fig. 3B).

Our results indicate no evidence of strain sharing of these MAGs among individuals at any body site during the training duration. Instead, strains were preserved within an individual trainee across time, highlighting strain-level resiliency within an individual’s microbiome. In a longitudinal study, Oh et al. (58) describe the strain-level stability of skin microbial communities with evidence of stable individual-specific signatures over time as demonstrated through single nucleotide variation analyses of a commensal skin species, *Propionibacterium acnes*. Thus, the lack of strain-level transmission between trainees, as observed here, suggests that convergence, as indicated by Bray-Curtis dissimilarity trends, was likely a result of adaptation at the level of individual microbiomes to external factors (e.g., shared environmental influences). This also suggests an absence of direct strain-level transmission of potential infectious risk factors between individuals within the congregate setting. The analysis is, however, limited to genomes that were successfully assembled with sufficient coverage for comparisons. Notably, genomes of *Staphylococcus aureus*, a species commonly involved in skin and soft tissue infections, were not recoverable and trackable across samples.

### Pathogen detection at different body sites

**Fig 4:**
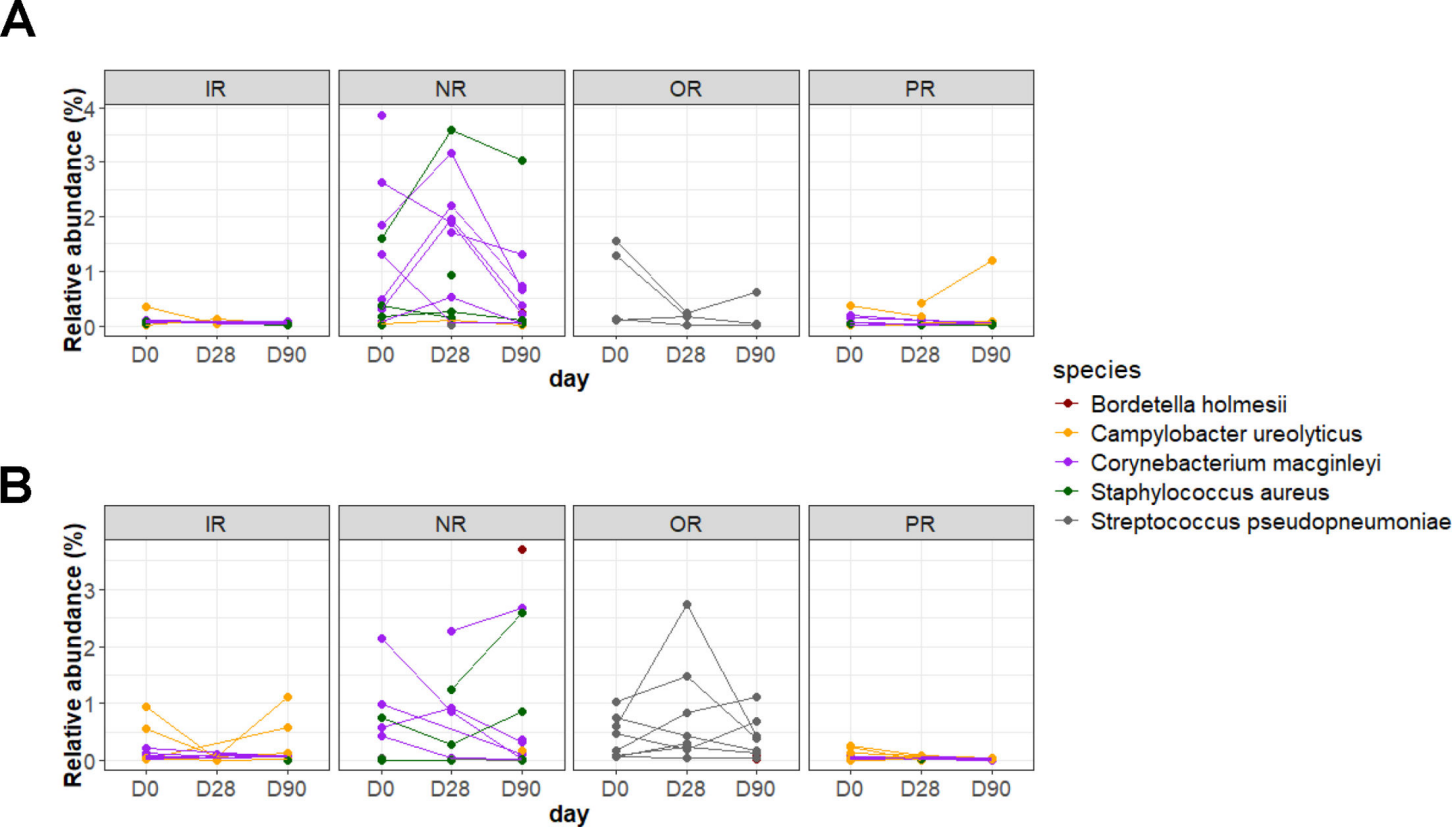
Pathogenic species identified from the TaxTriage pipeline in (**A**) platoon 2 and (**B**) platoon 4. Only species that had at least 0.01% relative abundance were considered. Four primary pathogens were identified: *Bordetella holmesii* (red)*, Campoylobacter ureolyticus* (orange), *Corynebacterium macginleyi* (purple), and *Streptococcus pseudopneumoniae* (gray). *Staphylococcus aureus* (green), a common skin and soft tissue infectious pathogens, was also identified. Lines connect samples that were collected from the same subject across time.

Due to the high occurrence of skin and soft-tissue infections in military personnel (4, 29), the presence of potential etiologic pathogens was evaluated using the TaxTriage pipeline that leverages a curated list of pathogens based on published literature for pathogen identification. TaxTriage identified and classified four species as primary pathogenic, across sampled sites, at relative abundances greater than 0.01% (Fig. 4). These include *Bordetella holmesii* (blood/respiratory pathogen), *Campylobacter ureolyticus* (gastrointestinal pathogen), *Corynebacterium macginleyi* (ocular pathogen), and *Streptococcus pseudopneumoniae* (respiratory/sputum pathogen).

Across both platoons, *S. pseudopenumoniae* was mainly detected in OR samples while *C. ureolyticus* and *C. macginleyi* were detected across IR, PR and NR samples (Fig. 4). Accordingly, a *C. ureolyticus* MAG was also recovered from the PR co-assembly (Fig. 3C). *B. holmesii* was detected in only three NR and two OR samples. While at low abundance, these organisms, when detected, appeared to be consistently present within an individual across time. While the role of these four bacterial species in the context of the skin microbiome and disease susceptibility is not clear, the acquisition of these species during vulnerable periods could result in disease and infection. *B. holmesii*, for example, has been primarily recognized as a respiratory pathogen with known associations with bacteremia and septicemia in both immunocompetent and immunocompromised individuals (59, 60).

Although classified as a commensal species by TaxTriage, the results did indicate the presence of *Staphylococcus aureus* in 11 NR samples (Fig. 4). Methicillin-resistant *S. aureus* have previously been described to be prevalent and involved in outbreaks within military settings (61, 62). Other skin and soft-tissue infection-causing species such as *Streptococcus pyogenes* and *Streptococcus dysgalactiae* were not detected. Overall, the presence of the risk factors indicated above could cause complications in the event of trauma and affiliated immune dysfunction (63). An improved understanding of microbial transmission of these and other etiologic agents of military-relevant disease, along with implementation of microbiome monitoring strategies in military trainee populations, could help minimize infection risks and reduce operational health burdens.

When combined with environmental surveillance, such studies that assess patterns in microbiome convergence and microbial sharing can provide valuable insights into how the built environment shapes microbiome exposures in both military and civilian populations. These findings can inform the design of shared spaces such as schools, hospitals, and offices where ventilation systems and hygiene practices can influence community-level microbial transmission (64). In hospital settings, where convergence can facilitate the spread of pathogenic or drug-resistance microbes, this foundational knowledge is essential for developing more effective sanitation protocols. Moreover, this can lead to interventions involving controlled microbial exposure such as the use of probiotics to promote beneficial microbial convergence and improve overall health and well-being.

### Conclusion

In this study, we assessed microbiome composition and convergence at different host body sites in a military trainee population where diet, routines, physical activities, and living conditions are constrained. We described preliminary evidence of local environment-dependent convergence across different body sites during the training duration, likely due to a combination of shared environmental factors and lifestyle practices. Nevertheless, our study is limited by both the sample size and the relatively narrow demographic distribution. As human microbiomes are influenced by ethnicity and genotypic backgrounds, these constraints reduce the generalizability of our findings to broader military and civilian populations. Future studies with increased longitudinal resolution and sampling of a wider range of body sites and more extensive breadth of demographic backgrounds will further elucidate microbial patterns and dynamics in a congregate environment.

Collectively, we demonstrated the feasibility of using shotgun metagenomic sequencing on low biomass samples to recover microbial features and metagenome-assembled genomes, which can be employed for comparative phylogenetics. This enables tracking and utilization of health-relevant, strain-level microbial features in a military population. The foundational data we present here on microbiome convergence and stability may guide the design of microbiome-based interventions targeted for military health and readiness needs.

## MATERIALS AND METHODS

### Study design and sample collection

Specimens were collected as previously described (3). Briefly, trainees were recruited from U.S. Army Infantry training at Fort Benning, Georgia. Individuals participated in a 14 week training cycle, composed of 200 trainees across four platoons. Swabs were obtained from nasal, oral, perianal, and inguinal regions at arrival (day 0), mid-training (day 28), and study conclusion (day 90). Specimens from 16 individuals across two platoons were selected for sequencing and analysis. The 16 individuals were all males, median age of 18 years (interquartile range: 18–19 years), and all originally from states in the southern United States. Fifteen individuals were non-Hispanic white, and one was African-American. This study was performed according to all relevant guidelines and regulations governing the performance of human subjects research and informed consent and was approved by the Uniformed Services University and Lawrence Livermore National Laboratory Institutional Review Boards.

### DNA extraction

Swab tips and buffer were centrifuged to sediment biomaterial. Buffer ATL (Qiagen; Germantown, Maryland) was added followed by pulse vortexing (10×) and shaking (10 min, 56°C, 600 RPM). Buffer volume was transferred to a lysing matrix A tube and processed via the FastPrep-24 (MP Biomedicals; Irvine, California) for 40 s at 6.0 m/s. Nucleic acid was purified from the supernatant using the QIAamp UCP Pathogen Mini kit (Qiagen; Germantown, Maryland) according to the manufacturer’s instructions. Extracted DNA was examined for quality and quantity via the Qubit HS dsDNA kit (Thermo Fisher; Foster City, California).

### Library preparation and sequencing

DNA libraries were prepared for sequencing using the Nextera DNA Library Preparation Kit (Illumina; San Diego, California). Quality and fragment size were assessed on the Agilent Tapestation 4200 (Agilent Technologies). Libraries were quantified using the Qubit HS dsDNA kit (Thermo Fisher; Santa Clara, California) and normalized to equivalent DNA quantities, pooled, and diluted according to the manufacturer’s standard recommendations. Shotgun metagenomic sequencing was performed using the Illumina NextSeq 500 with the NextSeq Series High Output Kit v2 (Illumina; San Diego, California), using 150 base pair paired-end reads.

### Sequence data processing and metagenomic classification

Raw sequence data were trimmed and quality filtered via fastp (65) using the following parameters; a quality Phred score of Q15, an unqualified base percentage of 40%, and a minimum read length of 15 base pairs. Minimap2 (66) was used to align reads to the GRCh38 human reference genome for *in silico* depletion of human reads. Taxonomic classification was performed using Centrifuge (67) and Recentrifuge (68) with a minimum hit length of 40 against a reference index constructed from National Center for Biotechnology Information nucleotide database (Jan 2023; [69]).

### Microbiome analysis and statistical tests

Taxonomic count tables were imported into phyloseq (70) in R (version 4.4.1) for downstream analysis. Alpha diversity was evaluated using Shannon and Simpson diversity indices, and beta diversity was determined using Bray-Curtis dissimilarity distances. PERMANOVA was carried out with the vegan package (71) to identify significant beta diversity variables and to estimate the contribution of each variable to compositional variability. Differentially abundant taxa at the species level was determined using DESeq2 (72). Wilcoxon rank-sum tests were used for bivariate comparisons. Subsequent Bonferroni adjustments were used for pairwise comparisons and FDR adjustments for multiple comparisons. TaxTriage (73) was used to identify primary pathogens present on swab samples.

### Metagenome assembly, binning, and strain tracking

Metagenome co-assemblies were carried out using MEGAHIT (74) for all body sites. MAGs were generated using MetaBinner (75) and genome bins were dereplicated using dRep (76). Taxonomy was assigned to each MAG using gtdbtk classify from the Genome Taxonomy Database Toolkit (GTDB-Tk) (77). Strain-level variant analysis was carried out using inStrain (57). A bootstrapped maximum likelihood phylogenetic tree was built using FastTree (78) with a multiple sequence alignment of MAGs that was created using gtdbtk align (77).

## Data Availability

The shotgun metagenomic sequencing data generated in this study have been deposited in the NCBI Sequence Read Archive (SRA) under BioProject accession number PRJNA1355317. To safeguard the privacy of military service member participants, host-derived reads were removed prior to deposition by aligning sequences to the human reference genome (GRCh38) and filtering out reads classified under the order Primates.

## SUPPLEMENTAL MATERIAL

Figure S1 spectrum.00474-25-s0001.pdf Microbiome composition and diversity.

Table S1 spectrum.00474-25-s0002.csv Per-sample taxonomic counts.

ASM does not own the copyrights to Supplemental Material that may be linked to, or accessed through, an article. The authors have granted ASM a non-exclusive, world-wide license to publish the Supplemental Material files. Please contact the corresponding author directly for reuse.
