# Supplementary material for: Microbiome dynamics in the congregate environment of U.S. Army Infantry training
Source: Microbiol Spectr. 2025 Dec 17;14(2):e00474-25. doi: 10.1128/spectrum.00474-25 (PMC12889126; doi:10.1128/spectrum.00474-25)
Supplement: Figure S1 — Microbiome composition and diversity. [file spectrum.00474-25-s0001.pdf]

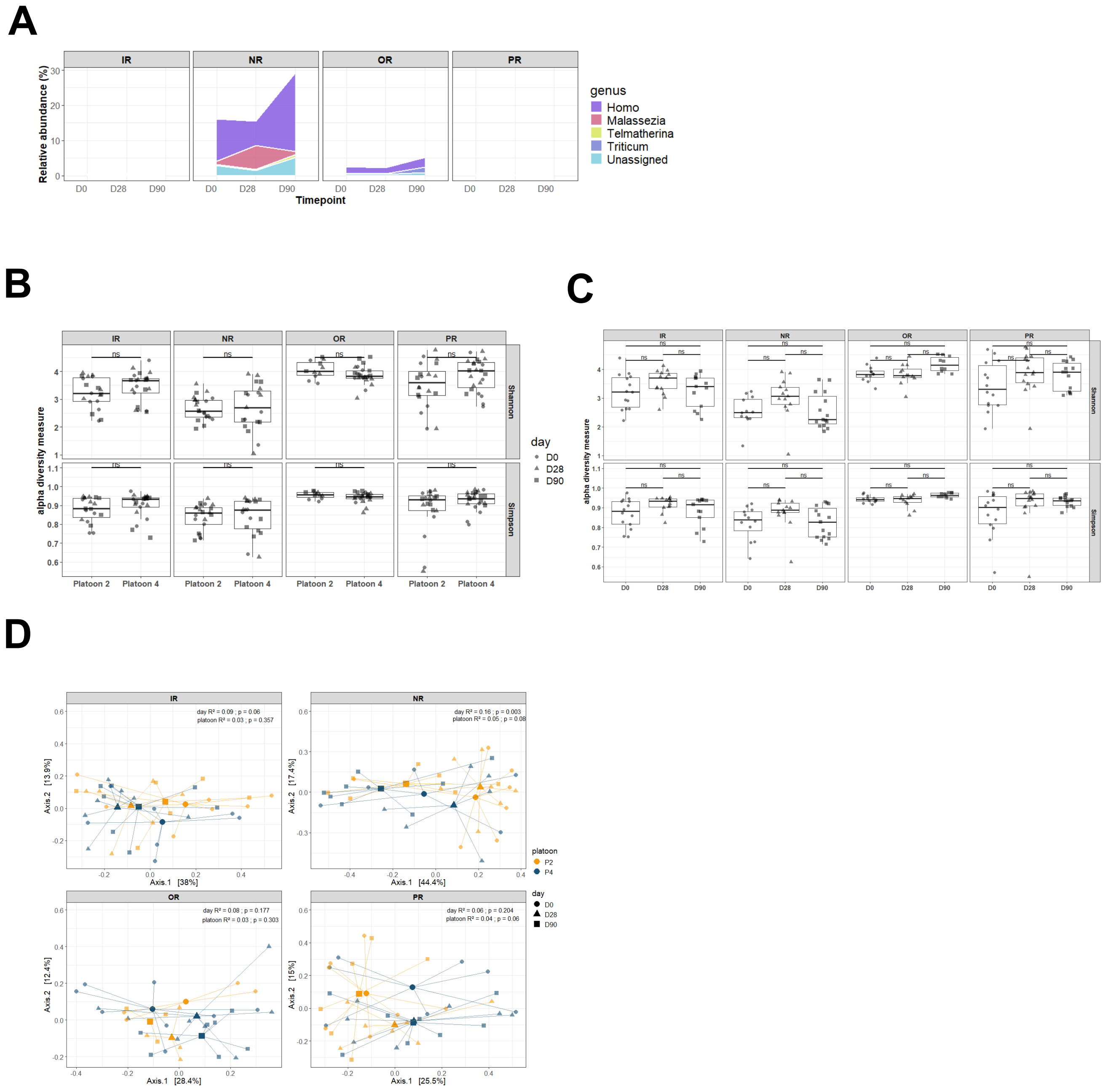

Figure S1. Microbiome composition and diversity. A) Relative abundances of classified eukaryotic reads. B) Alpha diversity comparisons between platoons stratified according to host body site. C) Alpha diversity comparisons between timepoints stratified according to host body site. D) PCoA representation of Bray-Curtis dissimilarity distances of samples within each body site. PERMANOVA analysis was carried out to determine significant differences between timepoints and platoons for each body site.
